# Clean cut to curtail respiration: OMA1 cleaves AIFM1 to tune stress-induced bioenergetics

**DOI:** 10.1038/s44318-026-00787-z

**Published:** 2026-04-30

**Authors:** Muhammad Irfan Afridi, Agnieszka Chacinska

**Affiliations:** IMol Polish Academy of Sciences, Warsaw, Poland

**Keywords:** Membranes & Trafficking, Metabolism, Organelles

## Abstract

Recent work reports integration of mitochondrial stress and cellular energetics by metalloprotease OMA1-mediated AIFM1 cleavage.

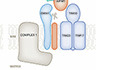

## Teaser

The mitochondrial peptidase OMA1 is a key responder to cellular stress, yet the molecular mechanisms by which it coordinates metabolic adaptations remain poorly defined. A recent study by Nishigori et al, (2026) identifies intermembrane space protein AIFM1 as a stress-dependent OMA1 substrate, whose cleavage results in dislocation from the inner mitochondrial membrane, inhibition of respiratory metabolism and cell growth.

Mitochondria maintain their integrity under cellular stress through inner membrane proteolytic quality control machineries. The stress-activated mitochondrial inner membrane peptidase OMA1 senses dysfunction via signals such as membrane depolarization and oxidative damage (Deshwal et al, 2020; Li et al, 2025). While OMA1 is well established to regulate mitochondrial fragmentation via OPA1 cleavage (Baker et al, 2014) and to initiate integrated stress response via DELE1 processing (Fessler et al, 2020; Guo et al, 2020), emerging evidence suggests that OMA1 targets additional substrates involved in mitochondrial bioenergetics and proteostasis (Krakowczyk et al, 2024).

**Figure 1:**
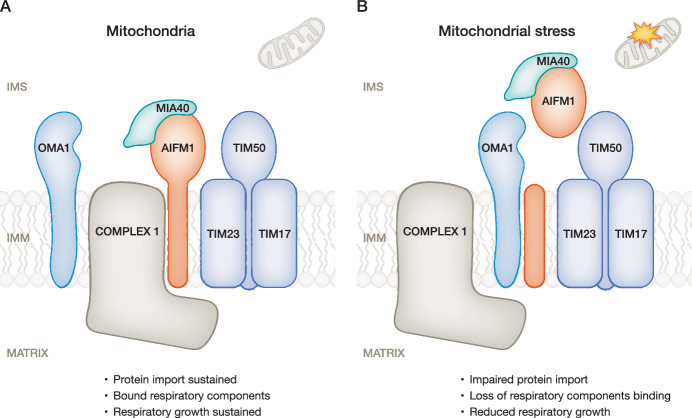
OMA1-mediated AIFM1 cleavage orchestrates mitochondrial homeostasis and stress signaling. (**A**) Under basal conditions, AIFM1 is localized at the inner mitochondrial membrane, where it associates with the TIM23 translocase and respiratory complex subunits, including Complex I. (**B**) Upon mitochondrial stress, OMA1 executes site-specific proteolysis of AIFM1, triggering its dissociation from the assemblies involving the TIM23 translocase.

In the current study, Nishigori et al, (2026) identified the membrane-anchored intermembrane space protein Apoptosis-inducing factor mitochondria-associated 1 (AIFM1) as an OMA1 substrate. AIFM1 mediates caspase-independent apoptosis and regulates oxidative phosphorylation as well as the activity of redox-dependent intermembrane space protein biogenesis pathway components, such as MIA40 (Salscheider et al, 2022; Wischhof et al, 2022). However, a complete characterization of AIFM1’s interaction partners and regulatory functions is currently lacking. Using a biochemical proteomics approach, Nishigori and team (2026) identified 38 proteins as the OMA1 interactome, of which AIFM1 showed strong enrichment. In a complementary approach, unique mitochondrial peptides exhibiting stress-dependent enrichment were characterized. In line, AIFM1 proteolytic fragments were specifically detected in OMA1-expressing but not depleted cells, confirming the role of OMA1 in mediating the AIFM1 cleavage during stress. Further, OMA1-mediated cleavage was found to release AIFM1 from the inner mitochondrial membrane into the intermembrane space, altering its spatial organization and interactions with mitochondrial machineries (Fig. 1). Notably, the kinetics of AIFM1 processing suggest selectivity in OMA1’s proteolytic activity, given that AIFM1 cleavage by OMA1 is delayed by minutes, whereas OMA1 cleaves OPA1 rapidly within minutes. This suggests temporal competition between OMA1 substrates, which is further evidenced by accelerated AIFM1 cleavage in OPA1-deficient cells, establishes a hierarchy of regulatory cleavages.

To differentiate the effects of AIFM1 dislocation from broader stress response, Nishigori et al, (2026) utilized a TEV protease system to bypass stress-dependent proteolytic cleavage by OMA1. Membrane-anchored AIFM1 integrates into heterogonous assemblies higher than 240 kDa. Upon cleavage and relocation from the membrane, AIFM1 shifts to a smaller 150 kDa, likely attributed to a dimeric state. Interestingly, OMA1 cleaves AIFM1 by a mechanism distinct from apoptotic AIFM1 processing (Polster et al, 2005) and the release of the AIFM1 due to OMA1 cleavage is not sufficient to trigger programmed cell death. One potential control factor for this interaction could be the mitochondrial oxidoreductase MIA40/CHCHD4. Under steady-state conditions, MIA40/CHCHD4 binds to AIFM1 in a dimeric structure, thereby increasing its folding stability (Salscheider et al, 2022; Brosey et al, 2025). The AIFM1-MIA40 complex functions as a sophisticated metabolic sensor for changes in NADH/NAD (Mussulini et al, 2025). High NADH/NAD ratio strengthens the interaction of AIFM1 with MIA40, conferring resistance to programmed cell death. Notably, AIFM1 variants unable to bind MIA40 become markedly destabilized and significantly more susceptible to OMA1-mediated cleavage. How the interplay between membrane dislocation caused by OMA1 and stabilization by MIA40 affects the ability of AIFM1 to be released from mitochondria and execute cell death remains to be investigated.

Surprisingly, however, in line with the direct interactions of membrane-embedded AIFM1 with the respiratory complexes subunits, Nishigori et al, (2026) further show that AIFM1 interacts with TIM23, a versatile and essential protein translocase involved in mitochondrial biogenesis. The TIM23 complex mediates translocation of precursor proteins across and into the inner membrane, playing a critical role specifically in OXPHOS subunit biogenesis (Chacinska et al, 2009). The findings of Nishigori and team (2026) suggest an exciting scenario, in which membrane-embedded AIFM1 contributes to the import of TIM23 import substrates under physiological conditions. The OMA1 cleavage-induced topologic transition of AIFM1 uncouples its function from the TIM23 protein import complex (Fig. 1). This has significant consequences to the mitochondrial proteome, resulting in lower abundance of 32 subunits of OXPHOS-related subunits, the majority of which could be attributed to the impaired protein import via TIM23. Thus, OMA1-mediated AIFM1 cleavage acts as a molecular switch that curtails an important auxiliary role of AIFM1 in mitochondrial respiratory protein biogenesis during stress, thereby inducing metabolic non-respiratory rewiring.

The finding that OMA1-mediated AIFM1 cleavage functions as a critical stress-responsive mechanism was also validated in two independent pathology models. First, in Cox10-deficient mice developing cardiomyopathy (Ahola et al, 2022), AIFM1 cleavage was shown to be essential for cardiac stress response in a manner strictly dependent on OMA1. Secondly, OMA1 also cleaves AIFM1 in response to viral stress in influenza-infected lungs, triggering a metabolic shift from OXPHOS to glycolysis, while simultaneously impairing mitochondrial protein import.

Together, Nishigori et al, (2026) introduce OMA1-dependent cleavage of AIFM1 as a conserved cellular stress response mechanism to induce topological switching that displaces AIFM1 from the mitochondrial inner membrane (Fig. 1). This displacement results in a decreased protein import of the OXPHOS components by the TIM23 translocase complex. The remodeling of the mitochondrial proteome caused by the OMA1-AIFM1 axis is a trigger for metabolic shifts under pathological conditions, resulting in inhibition of respiratory cell growth. Future research will need to focus on exploring the physiological functions of OMA1-dependent AIFM1 control. While the current study expands the role of AIFM1 beyond the previously characterized MIA40 pathway by finding an exciting link to TIM23, its precise function in import and biogenesis of mitochondrial proteins needs to be characterized mechanistically. Understanding the consequences of OMA1-mediated AIFM1 cleavage from the perspective of stress response falls into a second area of future investigation. Understanding how mitochondrial stress response mechanisms regulate protein biogenesis and lead to mitochondrial proteome alterations will provide critical insights into how cells coordinate metabolic adaptations and energy production.
